# Design, Synthesis and Docking Studies of Novel Macrocyclic Pentapeptides as Anticancer Multi-Targeted Kinase Inhibitors

**DOI:** 10.3390/molecules23102416

**Published:** 2018-09-20

**Authors:** Abd El-Galil E. Amr, Mohamed H. Abo-Ghalia, Gaber O. Moustafa, Mohamed A. Al-Omar, Eman S. Nossier, Elsayed A. Elsayed

**Affiliations:** 1 Pharmaceutical Chemistry Department, Drug Exploration & Development Chair (DEDC), College of Pharmacy, King Saud University, Riyadh 11451, Saudi Arabia; malomar1@ksu.edu.sa; 2 Applied Organic Chemistry Department, National Research Centre, 12622 Dokki, Giza, Egypt; 3 Department of Peptide Chemistry, National Research Centre, 12622 Dokki, Giza, Egypt; mhaboghalia@yahoo.com (M.H.A.-G.); gosman79@gmail.com (G.O.M.); 4 Department of Pharmaceutical Chemistry, Faculty of Pharmacy (Girls), Al-Azhar University, 11754 Cairo, Egypt; dr.emannossier@gmail.com; 5 Bioproducts Research Department, Zoology Department, Faculty of Science, King Saud University, Riyadh 11451, Saudi Arabia; eaelsayed@ksu.edu.sa; 6 Chemistry of Natural and Microbial Products Department, National Research Centre, 12622 Dokki, Cairo, Egypt

**Keywords:** macrocyclic pentapeptides, in vitro anticancer activity, multitarget, molecular modeling studies

## Abstract

A series of macrocyclic pyrido-pentapeptide candidates **2**–**6** were synthesized by using *N*,*N*-bis-[1-carboxy-2-(benzyl)]-2,6-(diaminocarbonyl)pyridine **1a**,**b** as starting material. Structures of the newly synthesized compounds were established by IR, ^1^H and ^13^C-NMR, and MS spectral data and elemental analysis. The in-vitro cytotoxicity activity was investigated for all compounds against MCF-7 and HepG-2 cell lines and the majority of the compounds showed potent anticancer activity against the tested cell lines in comparison with the reference drugs. Out of the macrocyclic pyrido-pentapeptide based compounds, **5c** showed encouraging inhibitory activity on MCF-7 and HepG-2 cell lines with IC_50_ values 9.41 ± 1.25 and 7.53 ± 1.33 μM, respectively. Interestingly, **5c** also demonstrated multitarget profile and excellent inhibitory activity towards VEGFR-2, CDK-2 and PDGFRβ kinases. Furthermore, molecular modeling studies of the compound **5c** revealed its possible binding modes into the active sites of those kinases.

## 1. Introduction

Cancer is still considered one of the most serious diseases threatening human life. In the past three decades, there enormous efforts have been undertaken to confront cancer diseases. Within such efforts, many therapeutic agents have been developed to treat cancer patients in their early, as well as late cancer-developing stages [1]. These agents vary from antibiotics, chemically synthesized compounds, and natural products-based drugs.

Macrocyclic motifs are commonly defined as a ring system containing 12 or more atoms [2]. They are privileged scaffolds in the fields of chemistry, biology, and medicine [3,4,5]. There are different classes of macrocycles like peptidic and nonpeptidic natural products, non-natural (synthetic) peptides and non-natural (synthetic) macrocycles [6].

**Figure 1:**
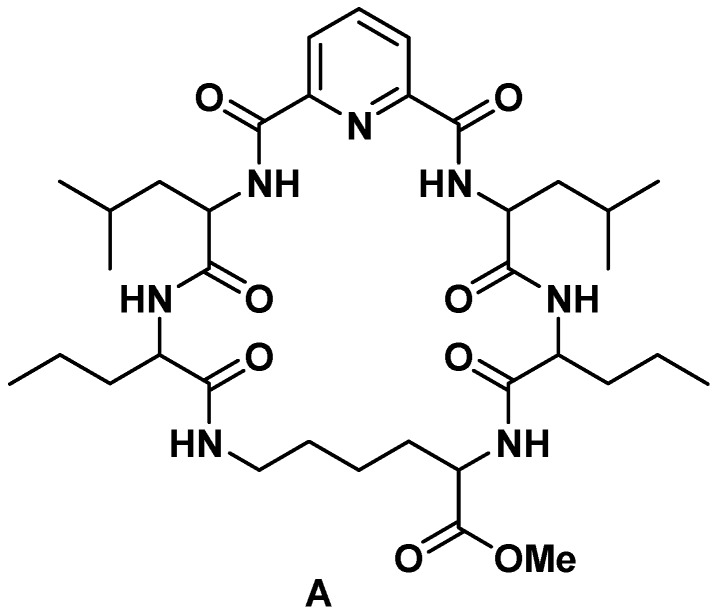
Structure of the anticancer agent.

Furthermore, peptides constitute a major class of important anticancer therapeutic agents [7]. Chemically synthesized peptides have been reported to exhibit antimicrobial, anti-inflammatory [8,9,10,11,12,13,14,15,16], as well as anticancer properties [17,18,19,20,21]. We have previously explored the analytical and biological characteristics of some bis-amino acid and peptide conjugates of dipicolinic acid [22]. Our studies of these compounds exemplified by compound [**A**] (Figure 1) revealed an interesting anticancer activity, probably via DNA intercalation, as well as an outstanding metal sensor property, particularly, for pollutant lead (Pb^2+^) cations [23].

**Figure 2:**
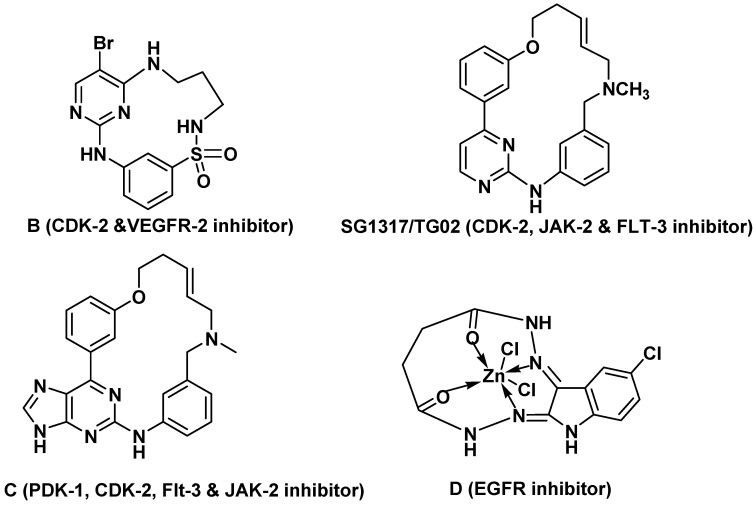
Reported and proposed macrocyclic conjugates with anticancer and different kinases inhibitory activity.

The advances in molecular biology and genetics help in identification of molecular targets that are related to cancer cells or overexpressed on them. The design of compounds affecting these targets improves the development of more selective anticancer drugs with less toxic side effects [24]. Macrocycles were reported to display antitumor properties, which may be attributed to inhibition of different enzymes involved in carcinogenesis cases. The prominent examples of macrocycles, illustrated in Figure 2, exhibited a potent inhibitory activity against various kinases, e.g., CDK-2, VEGFR-2, JAK-2, FLT-3, PDK-1 and EGFR [6,25,26,27,28].

In view of these observations and as continuation of our previous works in peptide heterocyclic chemistry, we have herein synthesized some new linear and macrocyclic peptidopyridine derivatives, and tested their anticancer activity in comparison to Tamoxifen and 5-Fluorouracil^®^ as positive controls. Furthermore, they were screened for their inhibitory activity against VEGFR-2, EGFR, PDGFRβ and CDK-2 enzymes. Additionally, molecular modeling study was performed to explore the most appropriate binding modes of the most potent target compounds.

## 2. Results and Discussion

### 2.1. Chemistry

**Scheme 1:**
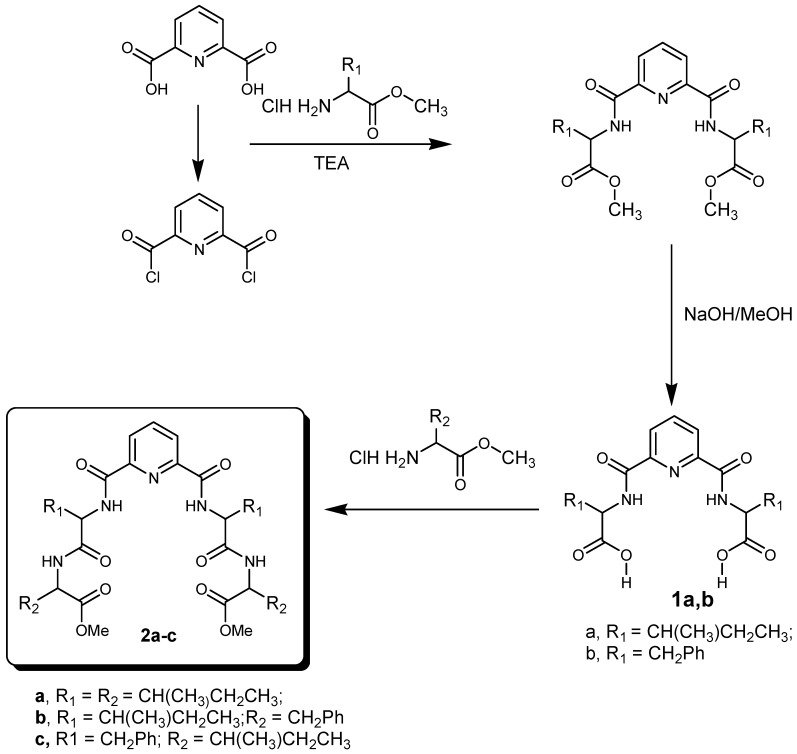
Synthetic pathway for compounds **2a**–**c.**

In the previous work [9,29], l-amino acid methyl esters was initially coupled with dipicolinic acid via the conventional acid chloride method to give the corresponding 2,6-bis-N^α^-l-diamino acid pyridine methyl ester derivatives. In the present work, a series of linear and macrocyclic pyridopentapeptide derivatives **2**–**6** were synthesized based on *N,N*-bis-[1-carboxy-2-(benzyl)]-2,6- (diamino-carbonyl)pyridine (**1a**,**b**) and they are screened as anticancer agents. Treatment of **1** with l-amino acid methyl ester hydrochloride in the presence of ethyl chloroformate in dichloromethane afforded the corresponding tetrapeptide pyridine methyl ester derivatives **2a**–**c**, respectively (Scheme 1).

**Scheme 2:**
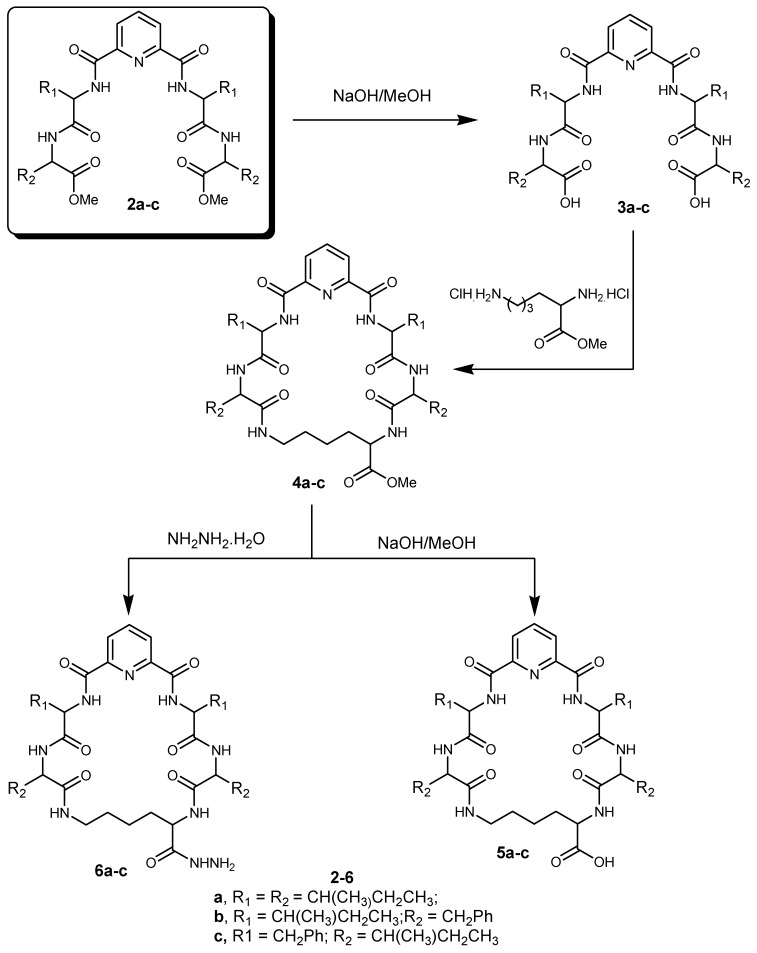
Synthetic pathway for compounds **3**–**6**.

Hydrolysis of **2a**–**c** with methanolic sodium hydroxide to afford the corresponding tetrapeptide pyridine derivatives **3a**–**c**, which were cyclized with l-lysine methyl ester by different methods to afford the corresponding cyclic pentapeptide esters **4a**–**c**, respectively. The cyclized pentapeptide esters **4a**–**c** was hydrolyzed with methanolic sodium hydroxide to give the corresponding cyclic pentapeptide acids **5a**–**c**, or by hydrazonolysis with hydrazine hydrate in methanol to give the corresponding cyclic pentapeptide acid hydrazides **6a**–**c**, respectively (Scheme 2).

### 2.2. Anticancer Activity

**Figure 3:**
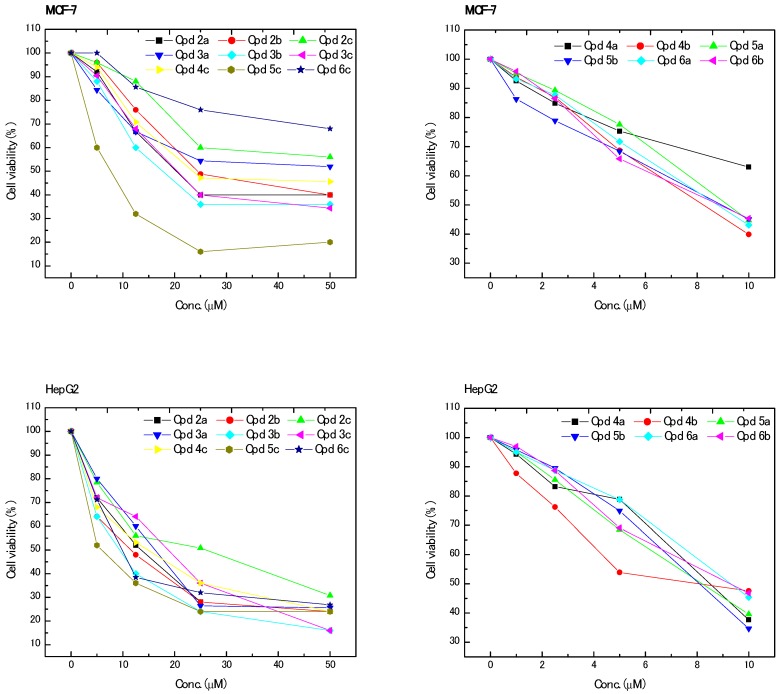
Effect of different concentrations of the prepared compounds on the viability of HepG-2 and MCF-7 cell lines. Data are expressed as means ± SEM (standard error mean).

The cytotoxic effects of all newly synthesized compounds were evaluated against MCF-7 and HepG-2 cell lines. Results obtained (Figure 3) showed that all prepared compounds affected both cell lines in a dose-dependent manner, where increasing the applied concentration gradually decreased cell viability. All tested compounds exhibited moderate to excellent cytotoxic activities on both tested cell lines in comparison with control drugs. Compounds **4a**, **4b**, **5a**, **5b**, **6a** and **6b** were more potent at lower range of tested concentrations. However, compounds **2c**, **3a**, **4a** and **6c** were considered inactive against MCF-7 cells, since they showed no practical IC_50_ (>100 µM). Concerning IC_50_ data (Table 1), it was noticed that only compounds **6a** and **6b** affected MCF-7 cells more than HepG-2 cells (IC_50_ = 11.83 ± 1.62 and 10.87 ± 1.10 µM for MCF-7, and 12.44 ± 1.3 and 11.53 ± 1.70 µM for HepG-2, respectively). While, the rest of the prepared compounds showed more noticeable anticancer activities in case of HepG-2 cells rather than MCF-7 cells. Comparing the obtained IC_50_ values for HepG-2 cells with those of the control drugs explored that compound **5c** was the most potent in comparison with tamoxifen and 5-fluorouracil (IC_50_ = 7.53 ± 1.33, 29.38 ± 1.15 and 43.84 ± 1.84 µM, respectively). Compounds **2a**, **2b**, **3b**, **4a**–**c**, **5a**–**c** and **6a**–**c** afforded higher cytotoxic activity, while compounds **3a** and **3c** gave approximately equipotent cytotoxic activity (IC_50_ = 26.01 ± 2.35 and 26.64 ± 1.85 µM, respectively). For MCF-7 cell lines, **5c** was also the most active derivative in comparison with tamoxifen (IC_50_ = 9.41 ± 1.25 and 22.40 ± 2.42 µM, respectively), followed by compounds **4b**, **6b**, **5b**, **6a** and **6a** (IC_50_ = 10.45 ± 1.33, 10.87 ± 1.10, 11.32 ± 1.15, 11.83 ± 1.62 and 12.67 ± 2.40 µM, respectively).

By analysis of the previous results, the cytotoxic activities of the tested compounds, with the exception of compound **4c**, were in the following order compound **5** > **6** > **4** > **3** > **2**. Concerning the structure-activity relationships of the synthesized compounds, it was observed that the open chain derivatives **2** and **3** have lower activity than the cyclized derivatives **4**–**6**. Furthermore, insertion of benzyl moiety at R_1_ adjacent to the pyridine scaffold in **2c** and **3c** gave a marked decrease in the cytotoxic activity compared to their series **2a**,**b** and **3a**,**b**, respectively. This decrease may be due to formation of steric hindrance. The enhanced anticancer activities of compounds **4**–**6** may be contributed to the cyclization of these compounds. Contrary to what was found in the open chain derivatives **2c** and **3c**, the increased aromaticity of the cyclopeptide by the location of the phenyl group of phenylalanine neighboring the pyridine nucleus may be the cause of improved activity of compound **5c** > **5b** > **5a**.

### 2.3. In Vitro Enzymatic Assays

**Figure 4:**
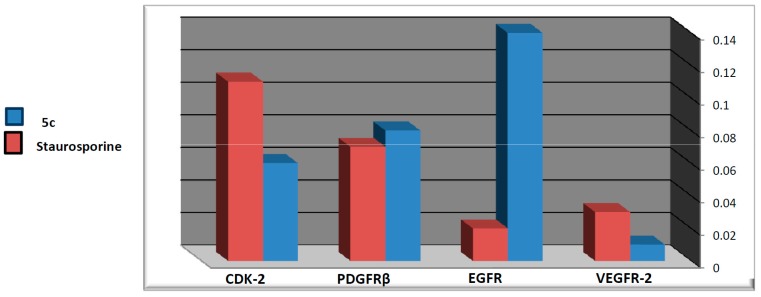
IC_50_ of compound **5c** against VEGFR-2, EGFR, PDGFRβ and CDK-2 kinases in comparison with staurosporine.

Upon cellular screening on MCF-7 and HepG-2, compound **5c** exhibited higher anticancer activity in comparison with tamoxifen and 5-fluorouracil. So, it was subjected for in vitro inhibition assessment against a panel of four different kinases; VEGFR-2, EGFR, PDGFRβ and CDK-2 kinases using staurosporine as multitarget inhibitor. As shown in Table 2 and Figure 4, compound **5c** exhibited three folds increase in the inhibitory effect against VEGFR-2 (IC_50_ = 0.01 ± 1.25 µM), two folds increase against CDK-2 (IC_50_ = 0.06 ± 1.27 µM) and approximately equipotent against PDGFRβ (IC_50_ = 0.08 ± 1.45 µM) in comparison with staurosporine (IC_50_ = 0.03 ± 1.10, 0.11 ± 1.13 and 0.07 ± 1.65 µM, respectively). Furthermore, the inhibitory activity of compound **5c** was moderate against EGFR in comparison with the reference (IC_50_ = 0.14 ± 1.00 and 0.02 ± 1.32 µM, respectively). These data suggested that compound **5c** is a promising multitarget kinase inhibitor.

### 2.4. Molecular Modeling Studies

The kinase inhibitory assay revealed that compound **5c** showed promising inhibitory activity against three kinases, namely VEGFR-2, CDK-2 and PDGFRβ. So, docking simulation was performed using Molecular Operating Environment (MOE^®^) 2008.10 [30,31] to predict the binding modes, affinities, and orientations of compound **5a** at the active sites of them. The X-ray crystallographic structure of PDGFRβ was not fully resolved [32]. On the other hand, the X-ray crystallographic structures were reported for VEGFR-2 (pdb code: 4ASD) [33] with sorafenib and for CDK-2 (PDB ID: 2J9M) [34] with PY8. So the docking study was achieved for both VEGFR-2 and CDK-2 kinases

**Figure 5:**
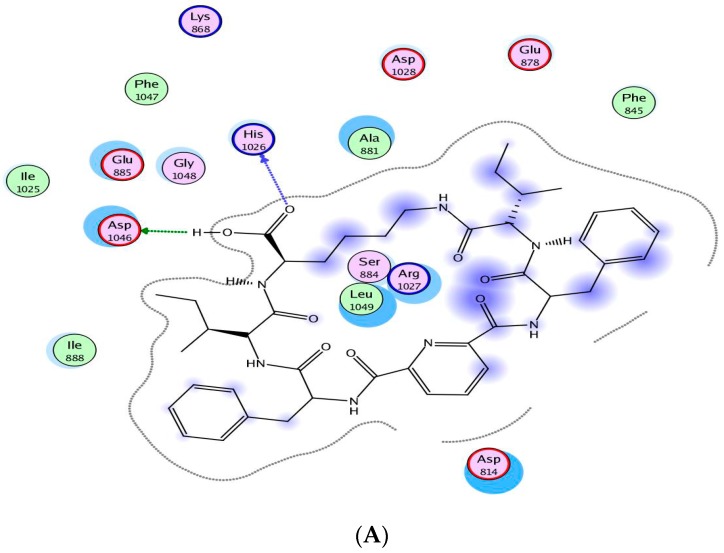

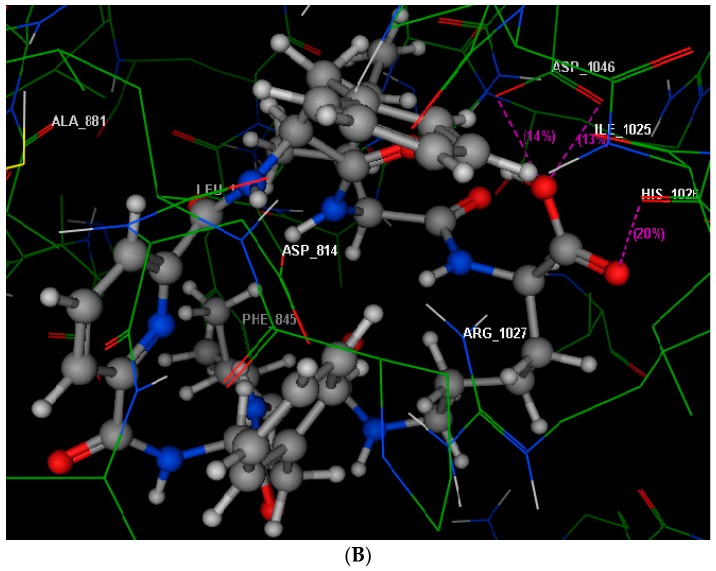
Docking solution for **5c** in the active site of VEGFR-2; (**A**,**B**) showing 2D and 3D ligand-receptor interactions (hydrogen bonds are illustrated as arrows; C atoms are colored gray, N blue and O red).

The binding model, shown in Figure 5, was exemplified by the interaction of compound **5c** with VEGFR-2. The carboxylic moiety of **5c** shared in the binding pattern with three hydrogen bond donors; one H-bond was between CO group and the backbone of His1026 (distance: 2.99 Å), and the others were between OH group and the sidechain of Asp1046 (distance: 1.92 and 2.65 Å). Moreover, the residues Ser884, Arg1027 and Leu1049 were inserted nicely inside the centre of cyclic pentapeptide scaffold.

**Figure 6:**
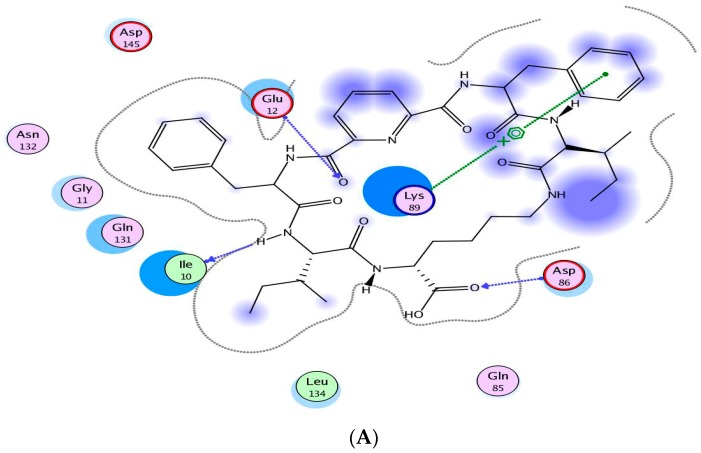

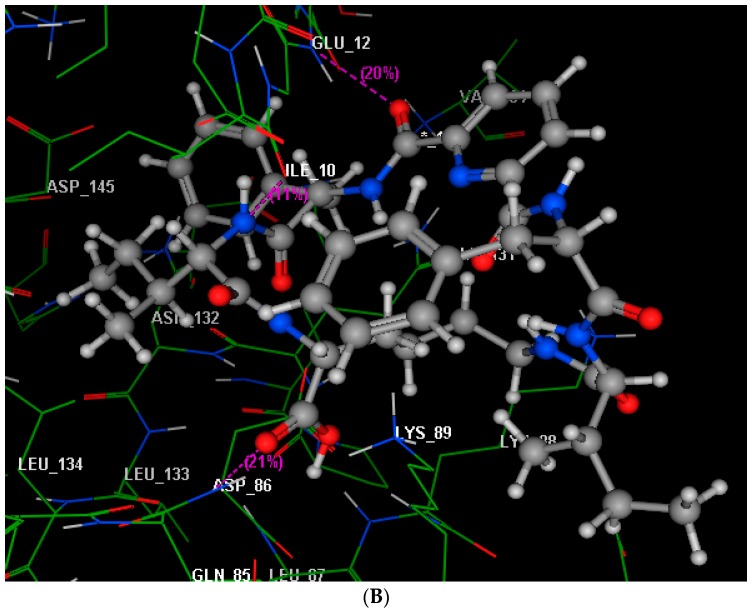
Docking solution for **5c** in the active site of CDK-2; (**A**,**B**) showing 2D and 3D ligand-receptor interactions (hydrogen bonds are illustrated as arrows; C atoms are colored gray, N blue and O red).

Interaction of compound **5c** with the binding site of CDK-2 kinase was illustrated in Figure 6. There was H-bond acceptor between CO of the carboxylic group and the backbone of Asp86 (distance: 2.89 Å). Another H-bond acceptor was established between the amide nitrogen and the backbone of Ile10 (distance: 1.80 Å). Furthermore, carbonyl group placed at p-2 of pyridine ring was linked to backbone of Glu12 (distance: 2.66 Å). In addition, Lys89 located in the centre of cyclic pentapeptide scaffold formed arene-cation interaction with centroid of benzyl ring.

Finally, it is deduced from molecular docking studies that the good fitting of compound **5c** in the active sites of VEGFR-2 and CDK-2 enzymes via different types of interactions could be attributed to the presence of carboxylic group and cyclic pentapeptide scaffold.

## 3. Materials and Methods

### 3.1. Chemistry

Melting points were determined in an “Electro Thermal” Digital melting point apparatus (Shimadzu, Tokyo, Japan), (model: IA9100). Elemental analysis was found within the acceptable limits of the calculated values (Microanalytical Unit, NRC). Infrared spectra (KBr) were recorded on a Nexus 670 FTIR Nicolet, Fourier Transform infrared spectrometer (Perkin Elmer, Hopkinton, MA, USA). Proton nuclear magnetic resonance (^1^H-NMR) spectra were run in [*d*_6_] DMSO on Jeol 270 MHz or 500 MHz instruments ((Tokyo, Japan). Chemical shifts d are given in ppm. Mass spectra were run on a MAT Finnigan SSQ 7000 spectrometer (Shimadzu, Kyoto, Japan; Model: QP2010 ultra), using the electron impact technique (EI). Analytical thin layer chromatography (TLC) was performed on silica gel aluminum sheets, 60 F254 (E. Merck). Specific optical rotations were measured with a A. Krawss, Optronic, P8000a polarimeter (A. Krüss, Hamburg, Germany), in a 1 dm length observation tube, at the indicated conditions, and according to the equation: [a]T D =100. a = (*c l*), where a = observed rotation angle; D = sodium line (l = 589 nm); *c* =concentration (g = 100 mL); *l* = path length in dm; and *T* = temperature (°C). The following solvent systems (by volume) were used as eluents for the development of the plates: S: chloroform-methanol-acetic acid (85:10:5); S_1_: S-petroleum ether (b.p. 40–60 °C) (1:1); S_2_: S-petroleum ether (b.p. 40–60 °C) (3:2); S_3_: S-petroleum ether (b.p. 40–60 °C) (1:2) and S_4_: butanol-water-acetic acid-pyridine (120:48:12:40). It is generally known that basic reaction media enhance racemization. However, under the reaction conditions employed in this work, especially short reaction times and temperatures below (0 °C), only negligible racemization was observed.

#### 3.1.1. Synthesis of N^α^-dipicolinoyl-bis[dipeptide methyl ester] Derivatives (**2a**–**c**)

Ethyl chloroformate (0:2 mL, 2 mmol) was added to a stirred and cold (−15 °C) dichloromethane solution (20 mL) of the corresponding N^α^-dipicolinoyl-bis[amino acid] (**1a**,**b**) (1 mmol), containing *N*-methylmorpholine (0:2 mL, 2 mmol). The reaction mixture was stirred for additional 10 min, then a cold dichloromethane solution (20 mL) of the free amino acid methyl esters, namely, l-isolucine-OMe or l-phenyalanine-OMe (2 mmol), was added. Stirring was maintained for 3 h at (−15 °C), then for 12 h at room temperature. The reaction mixture was washed with water, 1 N sodium bicarbonate, 1 N potassium hydrogen sulfate and water, and dried over anhydrous calcium chloride. The solvent was evaporated under reduced pressure to dryness, and the obtained oily residue was solidified by trituration with a dry ether-*n*-hexane mixture. The obtained solid was collected by filtration and crystallized from ethanol-*n*-hexane to give the corresponding 2,6-pyridine-bis-dipeptide ester derivatives (**2a**–**c**), respectively.

*N^α^-Dipicolinoyl-bis[l-ILe-l-ILe-methyl ester]* (**2a**). Yield: 80%; m.p. 106–108 °C, ${\left[\alpha \right]}_{D}^{25}$: −71 (C, 0.02, MeOH). IR (KBr, cm^−1^): ν = 3304 (NH stretching), 3070 (CH, aromatic), 2966 (CH, aliphatic), 1746 (C=O, ester), 1654 and 1440 (C=O amide I and II, respectively). ^1^H-NMR (500 MHz, ppm, DMSO-*d*_6_): δ = 8.54–8.51 (m, 3H, Pyr-H), 8.40–8.20 (s, 4H, 4NH, D_2_O exchangeable), 4.56 (d, 2H, *J* = 8.8 Hz, 2NHCH, l-Ile), 4.51 (d, 2H, *J* = 8.8 Hz, 2NHCH, l-Ile), 3.61 (s, 6H, 2OCH_3_), 3.32–3.30 (m, 4H, 4NHCHCH, l-Ile), 1.23–1.16 (m, 8H, 4CH_2_, l-Ile), 0.96–0.81 (m, 24H, 8CH_3_, l-Ile). ^13^C-NMR (125 MHz, ppm, DMSO-*d*_6_): δ = 171.6, 170.9 (2C, 2COOCH_3_), 162.8 (2C, 2CO), 148.7 (2CO, Pyridine dicarbonyl), 139.9 (2 C, pyridine, C_2_, C_6_), 130.3 (1C, pyridine, C_4_), 124.7 (2 C, pyridine, C_3_, C_5_), 56.7–54.5 (4 C, NHCH), 50.2 (2 C, 2OCH_3_), 40.3, 39.3 (4 C, 4NHCHCH, l-Ile), 39.0, 36.0 (4C, 2 CH_2_), 24.8, 11.0 (8 C, 8CH_3_, l-Ile). MS (EI, 70 eV): *m*/*z* (%) = 648 (M^+^ + 1, 3.62), 647 (M^+^, 3.74), 591 (18.87), 475 (100), 330 (16.79), 302 (14.78), 57 (1.16), 55 (0.53). Analysis for C_33_H_53_N_5_O_8_ (647.80): Calcd. C, 61.18; H, 8.25; N, 10.81. Found: C 61.15, H 8.22, N 10.77.

*N^α^-Dipicolinoyl-bis[l-ILe-l-Phe-methyl ester]* (**2b**). Yield: 70%; m.p. 122–124 °C, ${\left[\alpha \right]}_{D}^{25}$: −57 (C, 0.02, MeOH). IR (KBr, cm^−1^): ν = 3330 (NH stretching), 3087 (CH, aromatic), 2967 (CH, aliphatic), 1724 (C=O, ester), 1653 and 1532 (C=O amide I and II, respectively). ^1^H-NMR (500 MHz, ppm, DMSO-*d*_6_): δ = 8.57–8.53 (m, 3H, Pyr-H), 8.54 (s, 4H, 4NH, D_2_O exchangeable), 8.25–8.20 (m, 10H, Ar-H, l-Phe-ala), 4.56 (t, 2H, *J* = 5.02 Hz, 2NHCH, l-Phe-ala), 4.21 (d, 2H, *J* = 8.8 Hz, 2NHCH, l-Ile), 4.16 (d, 4H, *J* = 8.1 Hz, 2CH_2_, l-Phe-ala), 3.31 (s, 6H, 2 OCH_3_), 2.98–2.92 (m, 2H, 2NHCHCH, l-Ile), 1.43–1.36 (m, 4H, 2CH_2_, l-Ile), 0.96–0.81 (m, 12H, 4CH_3_, l-Ile). ^13^C-NMR (125 MHz, ppm, DMSO-*d*_6_): δ = 172.6 (2 C, 2COOCH_3_), 170.7, 162.8 (2CO, l-Ile), 148.7 (2CO, Pyridine dicarbonyl), 130.0 (2C, pyridine, C_2_, C_6_), 128.3 (1C, pyridine, C_4_), 125.7 (2 C, pyridine, C_3_, C_5_), 138.2, 127.5, 126.1, 125.4 (12C, Ph-C), 56.8, 56.3 (4C, NHCH), 52.9 (2C, 2OCH_3_), 40.4, 40.1 (2C, 2CH_2_, l-Phe-ala), 39.8–38.7 (2C, 2NHCHCH, l-Ile), 37.2–36.1 (2C, CH_2_), 24.7, 11.0 (4C, 4CH_3_, l-Ile). MS (EI, 70 eV): *m*/*z* (%) = 717 (M^+^ + 1, 0.85), 620 (1.95), 563 (7.83), 461 (100), 404 (3.59), 330 (30.42), 302 (43.45), 69 (8.42), 57 (3.89), 51 (0.96). Analysis for C_39_H_49_N_5_O_8_ (715.80): Calcd. C, 65.44; H, 6.90; N, 9.78. Found: C 65.42, H 6.88, N 9.76.

*N^α^-Dipicolinoyl-bis[l-Phe-l-ILe- methyl ester]* (**2c**). Yield: 65%; m.p. 150–152 °C, ${\left[\alpha \right]}_{D}^{25}$: −30 (C, 0.02, MeOH). IR (KBr, cm^−1^): ν = 3338 (NH stretching), 3100 (CH, aromatic), 3000 (CH, aliphatic), 1780 (C=O, ester), 1695 and 1600 (C=O amide I and II, respectively). ^1^H-NMR (500 MHz, ppm, DMSO-*d*_6_): δ = 8.90–8.85 (m, 3H, Pyr-H), 8.50–8.41 (s, 4H, 4NH, D_2_O exchangeable), 7.60–7.25 (m, 10H, Ar-H, l-Phe-ala), 4.55 (t, 2H, *J* = 5.02 Hz, 2NHCH, l-Phe-ala), 4.40 (d, 2H, *J* = 8.8 Hz, NHCH, l-Ile), 4.25 (d, 4H, *J* = 8.1 Hz, 2CH_2_, l-Phe-ala), 3.30–3.20 (s, 6H, 2OCH_3_), 2.90–2.70 (m, 2H, 2NHCHCH, l-Ile), 1.40–1.25 (m, 4H, 2CH_2_, l-Ile), 1.05–0.80 (m, 12H, 4CH_3_, l-Ile). ^13^C-NMR (125 MHz, ppm, DMSO-*d*_6_): δ = 174.0 (2CO, 2COOCH_3_), 171.9 (2CO, l-Phe-ala), 160.0 (2CO, Pyridine dicarbonyl), 150.5 (2C, pyridine, C_2_, C_6_), 138.0 (1C, pyridine, C_4_), 126.1 (2 C, pyridine, C_3_, C_5_), 139.2, 127.7, 126.4, 125.3 (12C, Ph-C), 61.0, 55.0 (4C, NHCH), 45.1 (2C, 2CH_2_, l-Phe-ala), 40.8, 40.5 (2C, 2NHCHCH, l-Ile), 39.0, 37.0 (2C, 2CH_2_), 24.0 (2C, 2OCH_3_), 18.0, 11.0 (4C, CH_3_, l-Ile). MS (EI, 70 eV): *m*/*z* (%) = 716 (M^+^, 18.00), 600 (22.12), 484 (34.00), 350 (29.39), 290 (32.67), 205 (75.81), 125 (100), 91 (82.32), 70 (33), 57 (45.87), 50 (18.85). Analysis for C_39_H_49_N_5_O_8_ (715.80): Calcd. C, 65.44; H, 6.90; N, 9.78. Found: C 65.40, H 6.83, N 9.77.

#### 3.1.2. Synthesis of N^α^-Dipicolinoyl-bis[dipeptide]derivatives (**3a**–**c**)

To a cold (−15 °C) solution of the corresponding tetrapeptide ester (**2a**–**c**) (1 mmol) in methanol (20 mL) with stirring, sodium hydroxide (1 N, 25 mL) was gradually added. The reaction mixture was stirred for 2 h at the same temperature then for 3 h at room temperature. The solvent was distilled off under reduced pressure, and the remaining aqueous solution was cooled and acidified with 1 N hydrochloric acid to pH = 3. The obtained solid was filtered off, washed with water, dried and crystallized from ethanol/water to give the corresponding tetrapeptide acids (**3a**–**c**), respectively.

*N^α^-Dipicolinoyl-bis[l-ILe-l-ILe]acid* (**3a**). Yield: 88%; m.p. 178–180 °C, ${\left[\alpha \right]}_{D}^{25}$: −101 (C, 0.02, MeOH). IR (KBr, cm^−1^): ν = 3280 (NH stretching), 3060 (CH, aromatic), 2964 (CH, aliphatic), 1748 (C=O, acid), 1651 and 1537 (C=O amide I and II, respectively). ^1^H-NMR (500 MHz, ppm, DMSO-*d*_6_): δ = 12.48 (s, 2 H, 2 OH, D_2_O exchangeable), 8.56–8.53 (m, 3H, Pyr-H), 8.39–8.22 (s, 4H, 4NH, D_2_O exchangeable), 4.52 (d, 4H, *J* = 8.8 Hz, 4NHCH, l-Ile), 3.61–3.58 (m, 4H, 4NHCHCH, l-Ile), 1.90–1.15 (m, 8H, 4CH_2_, l-Ile), 0.91–0.82 (m, 24H, 8CH_3_, l-Ile). ^13^C-NMR (125 MHz, ppm, DMSO-*d*_6_): δ = 171.5 (2C, 2COOH), 162.6 (2CO, l-Ile), 160.5 (2CO, Pyridine dicarbonyl), 139.7 (2C, pyridine, C_2_, C_6_), 137.0 (1C, pyridine, C_4_), 124.3 (2 C, pyridine, C_3_, C_5_), 56.9, 51.6 (4C, 4NHCH), 40.3, 39.2 (4C, 4NHCHCH, l-Ile), 38.9, 36.4 (4C, 4CH_2_, l-Ile), 23.8, 10.9 (8C, 8CH_3_, l-Ile). MS (EI, 70 eV): *m*/*z* (%) = 621 (M^+^ + 1, 9.35), 537 (20.12), 509 (100), 330 (25.60), 302 (49.23), 69 (17.34), 59 (52.77), 57 (18.36), 50 (0.92). Analysis for C_31_H_49_N_5_O_8_ (619.70): Calcd. C, 60.08; H, 7.97; N, 11.30. Found: C 60.08, H 7.92, N 11.29.

*N^α^-Dipicolinoyl-bis[l-ILe-l-Phe]acid* (**3b**). Yield: 82%; m.p. 184–186 °C, ${\left[\alpha \right]}_{D}^{25}$: −106 (C, 0.02, MeOH). IR (KBr, cm^−1^): ν = 3329 (NH stretching), 3072 (CH, aromatic), 2965 (CH, aliphatic), 1726 (C=O, acid), 1652 and 1532 (C=O amide I and II, respectively). ^1^H-NMR (500 MHz, ppm, DMSO-*d*_6_): δ = 12.65 (s, 2H, 2OH, D_2_O exchangeable), 8.52–8.43 (m, 3H, Pyr-H), 8.40–8.20 (s, 4H, 4NH, D_2_O exchangeable), 7.23–7.08 (m, 10H, Ar-H, l-Phe-ala), 4.45 (t, 2H, *J* = 5.02 Hz, 2NHCH, l-Phe-ala), 4.40 (d, 2H, *J* = 8.8 Hz, 2NHCH, l-Ile), 4.31 (d, 4H, *J* = 8.1 Hz, 2CH_2_, l-Phe-ala), 3.00–2.91 (m, 2H, 2NHCHCH, l-Ile), 1.9–1.5 (m, 4H, 2CH_2_, l-Ile), 1.15–0.8 (m, 12H, 4CH_3_, l-Ile). ^13^C-NMR (125 MHz, ppm, DMSO-*d*_6_): δ = 172.5 (2C, 2COOH), 169.1 (2C, 2CO, l-Ile), 162.6 (2C, 2CO, Pyridine dicarbonyl), 148.5 (2C, pyridine, C_2_, C_6_), 139.7 (1C, pyridine, C_4_), 126.00 (2 C, pyridine, C_3_, C_5_), 139.0, 127.2, 126.4, 125.6 (12C, Ph-C), 56.9, 53.2 (4C, 4NHCH), 40.3, 40.1 (2C, 2CH_2_, l-Phe-ala), 39.8, 38.7 (2C, 2NHCHCH, l-Ile), 37.0, 36.5 (2C, 2CH_2_, l-Ile), 23.8, 10.9 (4C, 4CH_3_, l-Ile). MS (EI, 70 eV): *m*/*z* (%) = 689 (M^+^ + 1, 1.78), 688 (M^+^, 4.41), 687 (8.50), 625 (71.56), 524 (100), 495 (92.50), 477 (51.54), 330 (56.17), 86 (36.66), 57 (12.05), 50 (2.34). Analysis for C_37_H_45_N_5_O_8_ (687.80): C, 64.61; H, 6.59; N, 10.18. Found: C 64.58, H 6.57, N 10.17.

*N^α^-Dipicolinoyl-bis[l-Phe-l-ILe]acid* (**3c**). Yield: 77%; m.p. 202–204 °C, ${\left[\alpha \right]}_{D}^{25}$: −70 (C, 0.02, MeOH). IR (KBr, cm^−1^): ν = 3321 (NH stretching), 3031 (CH, aromatic), 2967 (CH, aliphatic), 1725 (C=O, acid), 1655 and 1530 (C=O amide I and II, respectively). ^1^H-NMR (500 MHz, ppm, DMSO-*d*_6_): δ = 12.70 (s, 2 H, 2OH, D_2_O exchangeable), 9.60–8.50 (m, 3H, Pyr-H), 8.23–8.09 (s, 4H, 4NH, D_2_O exchangeable), 7.44–7.12 (m, 10H, Ar-H, l-Phe-ala), 4.90 (t, *J* = 5.02 Hz, 2H, 2NHCH, l-Phe-ala), 4.29 (d, 2H, *J* = 8.8 Hz, 2NHCH, l-Ile), 4.00 (d, 4H, *J* = 8.1 Hz, 2CH_2_, l-Phe-ala), 3.34–3.13 (m, 2H, 2NHCHCH, l-Ile), 1.23–1.16 (m, 4H, 2CH_2_, l-Ile), 0.92–0.80 (m, 12H, 4CH_3_, l-Ile). ^13^C-NMR (125 MHz, ppm, DMSO-*d*_6_): δ = 173.2 (2C, 2COOH), 172.6, 170.8 (2CO, l-Phe-ala), 164.9, 163.4 (2CO, Pyridine dicarbonyl), 148.6 (2C, pyridine, C_2_, C_6_), 139.6 (1C, pyridine, C_4_), 125.6 (2 C, pyridine, C_3_, C_5_), 138.15 126.8, 126.3, 125.8 (12C, Ph-C), 59.8, 56.4 (4C, 4NHCH), 43.1, 40.4 (2C, 2CH_2_, l-Phe-ala), 39.8, 39.2 (2C, 2NHCHCH, l-Ile), 39.0, 36.0 (2C, CH_2_), 24.7, 11.0 (4C, CH_3_, l-Ile). MS (EI, 70 eV): *m*/*z* (%) = 689 (M^+^ + 1, 2.05), 688 (M^+^, 4.22), 669 (8.33), 625 (22.60), 558 (67.30), 529 (49.57), 461 (18.79), 416 (26.29), 370 (100), 302 (11.74), 86 (29.02), 57 (15.14), 51 (5.33). Analysis for C_37_H_45_N_5_O_8_ (687.80): Calcd. C, 64.61; H, 6.59; N, 10.18. Found: C 64.60, H 6.60, N 10.18.

#### 3.1.3. Synthesis of Cyclo-(N^α^-Dipicolinoyl)-bis-[dipeptide]-l-Lys-OMe (cyclic pentapeptide methyl esters) (**4a**–**c**)

To a stirred and cold (−15 °C) dichloromethane solution (20 mL) of the corresponding N^α^-dipicolinoyl-bis[dipeptide] (**3a**–**c**) (1 mmol), containing *N*-methylmorpholine (0:2 mL, 2 mmol), ethyl chloroformate (0.2 mL, 2 mmol) was added and stirred for 20 min. To the reaction mixture, free l-lysine methyl ester (1 mmol) in dichloromethane (20 mL) was added at (−15 °C) with stirring. Stirring was maintained for 3 h, at (−15 °C), then for 12 h at room temperature. The reaction mixture was washed with water, 1 N sodium bicarbonate, 1 N potassium hydrogen sulfate and water, and then dried over anhydrous calcium chloride. The solvent was evaporated under reduced pressure to dryness, and the obtained oily residue was solidified by trituration with dry ether-*n*-hexane mixture. The crude product was purified by preparative thin layer chromatography using S3 as eluent to give the corresponding cyclic pentapeptide methyl esters (**4a**–**c**), respectively.

*Cyclo-(N^α^-dipicolinoyl)-bis-[l-ILe-l-ILe]-l-Lys-OMe* (**4a**). Yield: 78%; m.p. 77–79 °C, ${\left[\alpha \right]}_{D}^{25}$: −13 (C, 0.02, MeOH). IR (KBr, cm^−1^): ν = 3398 (NH stretching), 2945 (CH, aromatic), 2867, 2863 (CH, aliphatic), 1642 (C=O, ester), 1530, 1454 and 1396 (C=O amide I, II and III, respectively). ^1^H-NMR (500 MHz, ppm, DMSO-*d*_6_): δ = 8.90–8.76 (m, 3H, Pyr-H), 8.32, 8.21 (s, 6H, 6NH, D_2_O exchangeable), 4.66 (d, 4H, *J* = 8.8 Hz, 4NHCH, l-Ile), 4.45 (t, 1H, *J* = 9.7 Hz, CH, l-Lys), 3.84 (s, 3H, OCH_3_), 3.57–3.55 (m, 2H, CH_2_, NHCH_2_), 2.70–2.55 (m, 4H, 4NHCHCH, l-Ile), 2.00–1.45 (m, 6H, 3CH_2_, NHCH_2_CH_2_CH_2_CH_2_CHNH), 1.30–1.15 (m, 8H, 4CH_2_, l-Ile), 1.05–0.80 (m, 24H, 8CH_3_). MS (EI, 70 eV): *m*/*z* (%) = 743 (M^+^, 13.34), 568 (10.65), 460 (11.38), 129 (23.58), 96 (28.34), 85 (34.33), 69 (69.08), 60 (74.04), 57 (77.97), 55 (100), 50 (14.79). Analysis for C_38_H_61_N_7_O_8_ (743.90): Calcd. C, 61.35; H, 8.26; N, 13.18. Found: C 61.30, H 8.22, N 13.14.

*Cyclo-(N^α^-Dipicolinoyl)-bis-[l-ILe-l-Phe]-l-Lys-OMe* (**4b**). Yield: 81%; m.p. 104–106 °C, ${\left[\alpha \right]}_{D}^{25}$: −28 (C, 0.02, MeOH). IR (KBr, cm^−1^): ν = 3368 (NH stretching), 2945 (CH, aromatic), 2835 (CH, aliphatic), 1657 (C=O, ester), 1528, 1425 and 1222 (C=O amide I, II and III, respectively). ^1^H-NMR (500 MHz, ppm, DMSO-*d*_6_): δ = 8.60–8.58 (m, 3H, Pyr-H), 8.43, 8.21 (2s, 6H, 6NH, D_2_O, exchangeable), 7.22–7.10 (m, 10H, Ar-H, l-Phe-ala), 4.46 (t, 2H, *J* = 5.02 Hz, 2NHCH, l-Phe-ala), 4.44 (d, 2H, *J* = 8.8 Hz, 2NHCH, l-Ile), 4.43 (t, 1H, CH, l-Lys), 4.41 (d, 4H, *J* = 8.1 Hz, 2CH_2_, l-Phe-ala), 3.61–3.57 (m, 2H, CH_2_, NHCH_2_, l-Lys), 3.32 (s, 3H, OCH_3_), 3,00-2.95 (m, 2H, 2NHCHCH, l-Ile), 1.9–1.5 (m, 6H, 3CH_2_, NHCH_2_CH_2_CH_2_CH_2_CHNH), 1.4–1.35 (m, 4H, 2CH_2_, l-Ile), 0.91–0.81 (m, 12H, 4CH_3_, l-Ile). ^13^C-NMR (125 MHz, ppm, DMSO-*d*_6_): δ = 171.6 (COOCH_3_), 170.6, 162.7 (6C, 6CO), 148.5 (2C, pyridine, C_2_, C_6_), 139.8 (1C, pyridine, C_4_), 127.1 (2 C, pyridine, C_3_, C_5_), 138.8, 127.6, 126.8, 125.2 (12C, Ph-C), 56.9–51.7 (5C, 5NHCH), 40.34 (1C, NHCH_2_, l-Lys), 40.1 (2C, 2CH_2_, l-Phe-ala), 39.8, 39.5 (2C, 2NHCHCH, l-Ile), 39.22, 36.39 (5C, 5CH_2_), 23.2 (1C, OCH_3_), 15.3, 11.0 (4C, 4CH_3_, l-Ile). MS (EI, 70 eV): *m*/*z* (%) = 796 (M^+^, 1.05), 715 (9.49), 659 (5.53), 509 (71.40), 449 (9.59), 347 (26.17), 302 (100), 180 (62.98), 86 (48.45), 69 (30.62), 52 (0.14). Analysis for C_44_H_57_N_7_O_8_ (811.96): Calcd. C, 65.09; H, 7.08; N, 12.08. Found: C, 65.00; H, 7.00; N, 12.00.

*Cyclo-(N^α^-Dipicolinoyl)-bis-[l-Phe-l-ILe]-l-Lys-OMe* (**4c**). Yield: 75%; m.p. 140–142 °C, ${\left[\alpha \right]}_{D}^{25}$: −55 (C, 0.02, MeOH). IR (KBr, cm^−1^): *ν* = 3320 (NH stretching), 3065 (CH, aromatic), 2965 (CH, aliphatic), 1783 (C=O, ester), 1663, 1527 and 1447 (C=O amide I, II and III, respectively). ^1^H-NMR (500 MHz, ppm, DMSO-*d*_6_): δ = 8.80–8.78 (m, 3H, Pyr-H), 8.63, 8.51 (s, 6H, 6NH, D_2_O exchangeable), 7.52–7.10 (m, 10H, Ar-H, l-Phe-ala), 4.65 (t, 2H, *J* = 5.02 Hz, 2NHCH, l-Phe-ala), 4.50 (d, 2H, *J* = 8.8 Hz, 2NHCH, l-Ile), 4.40 (t, 1H, *J* = 9.7 Hz, CH, l-Lys), 4.36 (d, 4H, *J* = 8.1 Hz, 2CH_2_, l-Phe-ala), 3.82–3.74 (m, 2H, CH_2_, NHCH_2_, l-Lys), 3.52 (s, 3H, OCH_3_), 3,20–3.05 (m, 2H, 2NHCHCH, l-Ile), 1.85–1.45 (m, 6H, 3CH_2_, NHCH_2_CH_2_CH_2_CH_2_CHNH), 1.38–1.36 (m, 4H, 2CH_2_, l-Ile), 0.90–0.75 (m, 12H, 4CH_3_, l-Ile). ^13^C-NMR (125 MHz, ppm, DMSO-*d*_6_): δ = 174.2 (COOCH_3_), 172.8, 168.0 (6C, 6CO), 150.6 (2C, pyridine, C_2_, C_6_), 140.2 (1C, pyridine, C_4_), 125.6 (2 C, pyridine, C_3_, C_5_), 138.2 127.0, 126.2, 125.4 (12C, Ph-C), 60.3–55.9 (5C, 5NHCH), 48.40 (1C, NHCH_2_, l-Lys), 45.0 (2C, 2CH_2_, l-Phe-ala), 42.0, 41.5 (2C, 2NHCHCH, l-Ile), 40.20, 38.44 (5C, 5CH_2_), 25.5 (1C, OCH_3_), 13.3, 10.5 (4C, 4CH_3_, l-Ile). MS (EI, 70 eV): *m*/*z* (%) = 813 (M^+^ + 1, 25.06), 812 (M^+^, 42.13), 755 (33.32), 720 (37.79), 698 (22.27), 596 (21.32), 571 (30.36), 543 (32.52), 495 (20.68), 458 (25.09), 430 (56.12), 370 (100), 91 (14.24), 69 (10.40), 57 (3.84), 50 (3.80). Analysis for C_44_H_57_N_7_O_8_ (811.96). Calcd. C, 65.09; H, 7.08; N, 12.08. Found: C, 65.00; H, 7.00; N, 12.00.

#### 3.1.4. Synthesis of Cyclo-(N^α^-dipicolinoyl)-bis[dipeptide]-l-Lys-OH (cyclic pentapeptides) (**5a**–**c**)

To a stirred and cold methanolic solution (−5 °C, 20 mL) of the corresponding cyclic pentapeptide methyl ester (**4a**–**c**) (1 mmol), sodium hydroxide (1 N, 25 mL) was gradually added. The reaction mixture was stirred for 2 h at the same temperature, then for 3 h at room temperature. The solvent was distilled off under reduced pressure, and the remaining aqueous solution was cooled and acidified with 1 N hydrochloric acid to pH = 3. The obtained solid was filtered off, washed with water, dried and crystallized from ethanol-water to give the corresponding cyclic pentapeptides (**5a**–**c**), respectively.

*Cyclo-(N^α^-dipicolinoyl)-bis [l-ILe-l-ILe]-l-Lys-OH* (**5a**). Yield: 69%; m.p. 108–110 °C, ${\left[\alpha \right]}_{D}^{25}$: −21 (C, 0.02, MeOH). IR (KBr, cm^−1^): ν = 3317 (NH stretching), 3081 (CH, aromatic), 2967 (CH, aliphatic), 1724 (C=O, acid), 1655, 1533 and 1456 (C=O amide I, II and III, respectively). ^1^H-NMR (500 MHz, ppm, DMSO-*d*_6_): δ = 12.60 (s, 1H, OH, D_2_O exchangeable), 8.51–8.49 (m, 3H, Pyr-H), 8.46, 8.20 (2s, 6H, 6NH, D_2_O exchangeable), 4.70- (d, 4H, *J* = 8.8 Hz, 4 NHCH, l-Ile), 4.18 (t, 1H, *J* = 9.7 Hz, CH, l-Lys), 3.31–3.28 (m, 2H, CH_2_, NHCH_2_, l-Lys), 1.94–1.92 (m, 4H, 4NHCHCH), 1.90–1.88 (m, 6H, 3CH_2_, NHCH_2_CH_2_CH_2_CH_2_), 1.50–1.16 (m, 8H, 4CH_2_, l-Ile), 0.96–0.81 (m, 24H, 8CH_3_, l-Ile). MS (EI, 70 eV): *m*/*z* (%) = 730 (M^+^, 22.28), 682 (35.08), 621 (48.97), 565 (35.49), 395 (44.98), 285 (31.09), 244 (25.03), 69 (68.36), 57 (100), 51 (14.03). Analysis for C_37_H_59_N_7_O_8_ (729.90): Calcd. C, 60.88; H, 8.15; N, 13.43. Found: C 60.84, H 8.11, N 13.40.

*Cyclo-(N^α^-dipicolinoyl)-bis [l-ILe-l-Phe]-l-Lys-OH* (**5b**). Yield: 73%; m.p. 128–130 °C, ${\left[\alpha \right]}_{D}^{25}$: −35 (C, 0.02, MeOH). IR (KBr, cm^−1^): *ν* = 3312 (NH stretching), 3067 (CH, aromatic), 2965 (CH, aliphatic), 1725 (C=O, acid), 1655, 1531 and 1448 (C=O amide I, II and III, respectively). ^1^H-NMR (500 MHz, ppm, DMSO-*d*_6_): δ = 12.70 (s, 1H, OH, D_2_O exchangeable), 8.43–8.38 (3H, Pyr-H), 8.22, 8.20 (2s, 6H, 6NH, D_2_O exchangeable), 7.23–7.08 (m, 10H, Ar-H, l-Phe-ala), 4.45–4.42 (m, 5H, 5CH), 4.40 (t, 4H, *J* = 8.1 Hz, 2CH_2_, l-Phe-ala), 3.41–3.38 (m, 2H, NHCH_2_, l-Lys), 3,05–2.91 (m, 2H, 2NHCHCH, l-Ile), 1.95–1.50 (m, 6H, 3CH_2_, NHCH_2_CH_2_CH_2_CH_2_CHNH), 1.18 (m, 4H, 2CH_2_, l-Ile), 0.90–0.80 (m, 12H, 4CH_3_, l-Ile). ^13^C-NMR (125 MHz, ppm, DMSO-*d*_6_): δ = 172.6 (COOH), 170.4, 170.3 (4C, 4CO), 162.7 (2CO, Pyridine dicarbonyl), 148.6 (2C, pyridine, C_2_, C_6_), 139.7 (1C, pyridine, C_4_), 128.1 (2 C, pyridine, C_3_, C_5_), 139.0, 127.8, 126.7, 125.5 (12C, Ph-C), 57.0, 53.3 (5C, 5 NHCH), 40.4 (1C, NHCH_2_, l-Lys), 40.1, 39.8 (2C, 2CH_2_, l-Phe-ala), 39.5, 39.3 (2C, 2NHCHCH, l-Ile), 39.0, 36.5 (5C, 5CH_2_), 23.9, 11.0 (4C, 4CH_3_, l-Ile). MS (EI, 70 eV): *m*/*z* (%) = 797 (M^+^, 16.93), 708 (16.34), 621 (28.74), 562 (22.24), 504 (24.21), 321 (18.01), 281 (17.72), 170 (18.21), 85 (19.59), 71 (40.85), 61 (20.37), 59 (52.07), 57 (100), 51 (17.22). Analysis for C_43_H_55_N_7_O_8_ (797.90): Calcd. C, 64.72; H, 6.95; N, 12.29. Found: C 64.69, H 6.94, N 12.27.

*Cyclo-(N^α^-dipicolinoyl)-bis [l-Phe-l-ILe]-l-Lys-OH* (**5c**). Yield: 81%; m.p. 178–180 °C, ${\left[\alpha \right]}_{D}^{25}$: −30 (C, 0.02, MeOH). IR (KBr, cm^−1^): *ν* = 3327 (NH stretching), 3065 (CH, aromatic), 2965 (CH, aliphatic), 1721 (C=O, acid), 1659, 1527 and 1448 (C=O amide I, II and III, respectively). ^1^H-NMR (500 MHz, ppm, DMSO-*d*_6_): δ = 12.85 (s, 1H, OH, D_2_O exchangeable), 8.60–8.45 (m, 3H, Pyr-H), 8.33, 8.25 (2s, 6H, 6NH, D_2_O exchangeable), 7.55–7.10 (m, 10H, Ar-H, l-Phe-ala), 4.60–4.45 (m, 5H, 5CH), 4.35 (t, 4H, *J* = 8.1 Hz, 2CH_2_, l-Phe-ala), 3.50–3.45 (m, 2H, 1CH_2_, NHCH_2_, l-Lys), 3,25–3.10 (m, 2H, 2NHCHCH, l-Ile), 2.00–1.70 (m, 6H, 3CH_2_, NHCH_2_CH_2_CH_2_CH_2_), 1.30 (m, 4H, 2CH_2_, l-Ile), 1.10–0.70 (m, 12H, 4CH_3_, l-Ile). ^13^C-NMR (125 MHz, ppm, DMSO-*d*_6_): δ = 176.0 (COOH), 174.5, 172.8 (4CO, l-Phe-ala, l-Ile), 169.0 (2CO, Pyridine dicarbonyl), 150.3 (2C, pyridine, C_2_,C_6_), 140.9 (1C, pyridine, C_4_), 125.9 (2C, pyridine, C_3_, C_5_), 138.4, 127.0, 126.5, 125.7 (12C, Ph-C), 61.0, 56.3 (5C, 5NHCH), 45.5 (1C, NHCH_2_), 46.1, 40.8 (2C, 2CH_2_, l-Phe-ala), 39.5, 39.3 (2C, 2NHCHCH), 38.0, 36.5 (5C, 5 CH_2_), 24.5, 11.0 (4C, 4 CH_3_, l-Ile). MS (EI, 70 eV): *m*/*z* (%) = 797 (M^+^, 6.08), 681 (6.44), 636 (37.92), 621 (100), 588 (8.55), 565 (44.24), 304 (7.27), 89 (7.27), 67 (4.61), 57 (11.72), 55 (11.41). Analysis for C_43_H_55_N_7_O_8_ (797.90): Calcd. C, 64.72; H, 6.95; N, 12.29. Found: C 64.70, H 6.6.90, N 12.22.

#### 3.1.5. Synthesis of Cyclo-(N^α^-dipicolinoyl)-bis[dipeptide]-l-Lys-NHNH_2_ (cyclic pentapeptide hydrazides) (**6a**–**c**)

A mixture of cyclic pentapeptide methyl esters (**4a**–**c**) (1 mmol) and anhydrous hydrazine hydrate (0.35 mL, 10 mmol) in methanol (20 mL) was stirred at room temperature for 30 min., and then it was for 3 h. The reaction mixture was evaporated under reduced pressure; the obtained residue oil was solidified with ether, filtered off and crystallized from methanol/ether to afford the corresponding cyclic pentapeptide hydrazides (**6a**–**c**), respectively.

*Cyclo-(N^α^-dipicolinoyl)-bis[l-ILe-l-ILe]-l-Lys-NHNH*_2_ (**6a**). Yield: 60%; m.p. 180–182 °C, ${\left[\alpha \right]}_{D}^{25}$: −12 (C, 0.02, MeOH). IR (KBr, cm^−1^): *ν* = 3280 (NH stretching), 30,867 (CH, aromatic), 2964 (CH, aliphatic), 1644, 1532, 1450 and 1383 (C=O amide I, II, III and IV, respectively). ^1^H-NMR (500 MHz, ppm, DMSO-*d*_6_): δ = 9.02 (s, 1H, CONH, D_2_O exchangeable), 8.75–8.70 (3H, Pyr-H), 8.20, 8.10 (2s, 6H, 6NH, D_2_O exchangeable), 4.55 (d, 4H, *J* = 8.8 Hz, 4NHCH, l-Ile), 4.35–4.33 (m, 1H, CH, l-Lys), 4.20 (s, 2H, NH_2_), 3.70–3.65 (m, 2H, NHCH_2_, l-Lys), 2.55–2.40 (m, 4H, 4NHCHCH, l-Ile), 2.10–1.65 (m, 6H, 3CH_2_, NHCH_2_CH_2_CH_2_CH_2_CHNH), 1.40–1.25 (m, 8H, 4CH_2_, l-Ile), 1.15–0.75 (m, 24H, 8CH_3_, l-Ile). MS (EI, 70 eV): *m*/*z* (%) = 743 (M, 1.39), 709 (2), 656 (60.81), 616 (94.4), 584 (70.53), 543 (27.33), 515 (20.75), 471 (100), 443 (67.27), 358 (55.32), 302 (80.56), 86 (80.07), 69 (42.70), 57 (15.46), 50 (1.24). Analysis for C_37_H_61_N_9_O_7_ (743.90): Calcd. C, 59.74; H, 8.26; N, 16.95. *Found*: C 59.71, H 8.17, N 16.92.

*Cyclo-(N^α^-dipicolinoyl)-bis[l-ILe-l-Phe]-l-Lys-NHNH*_2_ (**6b**). Yield: 65%; m.p. 161–163 °C, ${\left[\alpha \right]}_{D}^{25}$: −40 (C, 0.02, MeOH). IR (KBr, cm^−1^): *ν* = 3289 (NH stretching), 3063 (CH, aromatic), 2964 (CH, aliphatic), 1650, 1529, 1448 and 1382 (C=O amide I, II, III and IV, respectively). ^1^H-NMR (500 MHz, ppm, DMSO-*d*_6_): δ = 9.12 (s, 1H, CONH, D_2_O exchangeable, Hydrazide), 8.53–8.51 (m, 3H, Pyr-H), 8.24, 8.19 (2s, 6H, 6NH, D_2_O exchangeable), 7.23–7.05 (m, 10H, Ar-H, l-Phe-ala), 4.50–4.48 (m, 6H, 2 NHCH, 2CH_2_, l-Phe-ala), 4.45–4.38 (m, 3H, 3CH), 4.35 (s, 2H, NH_2_), 3.32 (t, 2H, 2NHCH_2_, l-Lys), 2.79–2.75 (m, 2H, 2NHCHCH, l-Ile), 1.35–1.34 (m, 6H, 3CH_2_, NHCH_2_CH_2_CH_2_CH_2_CHNH), 1.23–1.20 (m, 4H, 2CH_2_, l-Ile), 0.90–0.60 (m, 12H, 4CH_3_, l-Ile). ^13^C-NMR (125 MHz, ppm, DMSO-*d*_6_): δ = 173.4, 173.0 (4C, 4CO, l-Phe-ala, l-Ile), 170.0 (1C, CO, Hydrazide), 164.5, 163.8 (2CO, Pyridine dicarbonyl), 150.7 (2C, pyridine, C_2_, C_6_), 142.9 (1C, pyridine, C_4_), 128.1 (2 C, pyridine, C_3_, C_5_), 139.0, 127.8, 126.7, 125.5 (12C, Ph-C), 60.6, 54.0 (5C, 5NHCH), 48.0 (1C, NHCH_2_, l-Lys), 44.4, 42.6 (2C, 2CH_2_, l-Phe-ala), 37.5, 37.0 (2C, 2NHCHCH, l-Ile), 34.0, 30.9 (5C, 5CH_2_), 24.2, 10.5 (4C, 4CH_3_, l-Ile). MS (EI, 70 eV): *m*/*z* (%) = 813 (M^+^+1, 2.42), 724 (13.09), 684 (40.82), 669 (30.09), 549 (37.88), 477 (55.84), 302 (100), 84 (24.44), 55 (22.15), 50 (3.04). Analysis for C_43_H_57_N_9_O_7_ (812.00): Calcd. C, 63.61; H, 7.08; N, 15.53. Found: C 63.59, H 7.02, N 15.47.

*Cyclo-(N^α^-Dipicolinoyl)-bis[l-Phe-l-ILe]-l-Lys-NHNH*_2_ (**6c**). Yield: 78%; m.p. 208–210 °C, ${\left[\alpha \right]}_{D}^{25}$: −66 (C, 0.02, MeOH). IR (KBr, cm^−1^): *ν* = 3282 (NH stretching), 3065 (CH, aromatic), 2963 (CH, aliphatic), 1645, 1524, 1447 and 1382 (C=O amide I, II, III and IV, respectively). ^1^H-NMR (500 MHz, ppm, DMSO-*d*_6_): δ = 9.35 (s, 1H, CONH, D_2_O exchangeable, Hydrazide), 9.10–8.90 (m, 3H, Pyr-H), 8.11, 8.09 (2s, 6H, 6NH, D_2_O exchangeable), 7.41–7.13 (m, 10H, Ar-H, l-Phe-ala), 4.82–4.80 (m, 6H, 2NHCH, 2CH_2_, l-Phe-ala), 4.25–4.00 (m, 3H, 3CH), 3.98 (s, 2H, NH_2_), 3.30–3.19 (m, 2H, NHCH_2_, l-Lys), 2.75–2.70 (m, 2H, 2NHCHCH, l-Ile), 1.4–1.32 (m, 6H, NHCH_2_CH_2_CH_2_CH_2_CHNH), 1.14–1.12 (m, 4H, 2CH_2_, l-Ile), 0.80–0.79 (m, 12H, 4CH_3_, l-Ile). ^13^C-NMR (125 MHz, ppm, DMSO-*d*_6_): δ = 174.6, 174.0 (4C, 4CO, l-Phe-ala, l-Ile), 172.0 (1C, CO, Hydrazide), 168.5, 166.9 (2C, 2CO, Pyridine dicarbonyl), 148.5 (2C, pyridine, C_2_, C_6_), 144.0 (1C, pyridine, C_4_), 126.1 (2C, pyridine, C_3_, C_5_), 138.5, 127.0, 126.7, 125.8 (12C, Ph-C), 61.0, 57.5 (5C, 5NHCH), 50.0 (1C, NHCH_2_, l-Lys), 47.4, 46.9 (2C, 2CH_2_, l-Phe-ala), 39.0, 38.8 (2C, 2CH, l-Ile), 36.0, 34.4 (5C, 5CH_2_), 27.7, 11.0 (4C, 4CH_3_, l-Ile). MS (EI, 70 eV): *m*/*z* (%) = 812 (M^+^, 0.8), 808 (2.6), 621 (1.00), 423 (1.2), 391 (1.2), 373 (1.8), 295 (3.6), 268 (11.9), 213 (11.3), 149 (17.2), 121 (26.9), 104 (36.2), 91 (100), 86 (43.4), 77 (35.8), 50 (24.2). Analysis for C_43_H_57_N_9_O_7_ (812.00): Calcd. C, 63.61; H, 7.08; N, 15.53. Found: C 63.58, H 7.07, N 15.49.

### 3.2. Anticancer Screening

In vitro bioassay on human cancer cell lines was adopted against two cancer cell lines (MCF-7 and HepG-2). HepG2, human hepatocellular carcinoma and MCF-7, human breast cancer cell lines were obtained from Sigma-Aldrich Chemie GmbH, Taufkirchen, Germany. Tamoxifen and 5-fluorouracil were used as reference standards according to a previously reported method [35,36,37,38,39].

### 3.3. In Vitro Enzymatic Assays

The in vitro enzyme inhibition determination for compound **5c** was carried out in confirmatory diagnostic unit, Vacsera, Egypt. The evaluation performed profiling of the compound **5c** against a range of four protein kinases [VEGFR-2, EGFR, PDGFRβ and CDK-2] by ELISA assay method using staurosporine as a reference according to the previously reported methods [40,41,42].

### 3.4. Molecular Modeling Studies

To evaluate the inhibitory activity and confirm best interactions between compound **5c** and target proteins, molecular docking studies were performed with the help of Molecular Operating Environment (MOE^®^) 2008.10 [30,31]. The three-dimensional X-ray structures of VEGFR-2 (PDB code: 4ASD) [33] and CDK-2 (PDB code: 2J9M) [34] were obtained from the Protein Data Bank through the internet.

## 4. Conclusions

In summary, a series of macrocyclic derivatives bearing pyrido-pentapeptide moiety were designed and synthesized from *N*,*N*-bis-[1-carboxy-2-(benzyl)]-2,6-(diaminocarbonyl)pyridine. Two human cancer cell lines (MCF-7 and HepG-2) were used to evaluate the anticancer potency of all synthesized compounds. Compared with tamoxifen, compounds **4a**, **4b**, **5a**, **5b**, **5c**, **6a**, **6b**, **6c** exhibited excellent potency against one or both cell lines. Furthermore, compound **5c** showed the strongest cytotoxic activities against both cell lines (IC_50_ = 9.41 ± 1.25 and 7.53 ± 1.33 μM, respectively) in comparison with tamoxifen and 5-fluorouracil. On the other hand, enzyme inhibition assay of compound **5c** against VEGFR-2, CDK-2 and PDGFRβ demonstrated promising inhibitory activity with IC_50_ 0.01 ± 1.25, 0.06±1.27 and 0.08±1.45 µM, respectively compared to staurosporine. Molecular docking study showed the ability of the compound to fit into the active sites of these enzymes with the best binding modes. It is worthy to mention macrocyclic pyrido-pentapeptide based compounds serve as a useful template for the further development of the anticancer multi-targeted agents.

## Figures and Tables

**Table 1:** IC_50_ of the tested compounds **2**–**6** against MCF-7 and HepG-2 cell lines.

Compound	IC_50_ (Mean ± SEM) (µM)	
	MCF-7	HepG-2
**2a**	31.64 ± 1.30	20.37 ± 1.36
**2b**	32.58 ± 1.50	15.80 ± 1.66
**2c**	-	35.52 ± 1.83
**3a**	-	26.01 ± 2.35
**3b**	25.33 ± 1.18	13.54 ± 1.45
**3c**	29.55 ± 2.06	26.64 ± 1.85
**4a**	-	11.59 ± 2.70
**4b**	10.45 ± 1.33	10.25 ± 2.20
**4c**	29.15 ± 1.39	18.84 ± 1.47
**5a**	12.67 ± 2.40	11.19 ± 1.95
**5b**	11.32 ± 1.15	10.09 ± 2.05
**5c**	9.41 ± 1.25	7.53 ± 1.33
**6a**	11.83 ± 1.62	12.44 ± 1.3
**6b**	10.87 ± 1.10	11.53 ± 1.70
**6c**	-	12.07 ± 1.68
Tamoxifen	22.40 ± 2.42	29.38 ± 1.15
5-Fluorouracil^®^	-	43.84 ± 1.84

IC_50_: Compound concentration required to inhibit the cell viability by 50%, SEM = standard error mean; each value is the mean of three values.

**Table 2:** Inhibitory evaluation of compound **5c** against VEGFR-2, EGFR, PDGFRβ and CDK-2 kinases.

Kinase	IC_50_ (Mean±SEM) (µM)	
	5c	Staurosporine
VEGFR-2	0.01 ± 1.25	0.03 ± 1.10
EGFR	0.14 ± 1.00	0.02 ± 1.32
PDGFRβ	0.08 ± 1.45	0.07 ± 1.65
CDK-2	0.06 ± 1.27	0.11 ± 1.13

IC_50_: Compound concentration required to inhibit the enzyme activity by 50%, SEM = Standard error mean; each value is the mean of three values.
